# A Scoping Review of Sensor-Based Capture of Eating and Drinking Occasions That Could Be Used for Enhancing Personalized Nutrition Interventions in Real Time

**DOI:** 10.1016/j.advnut.2025.100575

**Published:** 2025-12-20

**Authors:** Leanne Wang, Margaret Allman-Farinelli, Eric Hekler, Anna Rangan

**Affiliations:** 1 Discipline of Nutrition and Dietetics, Susan Wakil School of Nursing and Midwifery, Faculty of Medicine and Health, The University of Sydney, Sydney, New South Wales, Australia; 2 Charles Perkins Centre, The University of Sydney, Sydney, New South Wales, Australia; 3 Herbert Wertheim School of Public Health and Human Longevity Science, University of California San Diego, San Diego, CA, United States; 4 Center for Wireless and Population Health Systems, Qualcomm Institute, University of California San Diego, San Diego, CA, United States

**Keywords:** wearable sensor, wearable devices, sensor-based devices, nutrition care, personalized nutrition, precision nutrition, N-of-1, dietary assessment, just-in-time intervention, machine learning

## Abstract

**Background:**

Traditional dietary assessment methods used in nutrition research and practice are self-reported, burdensome, and prone to error, limiting utility. In recent years, sensor-based devices and machine learning approaches have emerged as promising tools for automating eating behavior detection and initiating different approaches to assessing intake. These technologies have potential to enhance dietary assessment and its accuracy, support personalized dietary interventions through real time, context-aware feedback, and reduce burden on respondents and practitioners. A prior 2021 review by the authors concluded that existing devices are not yet feasible for dietetic practice.

**Objectives:**

This study aims to conduct a scoping review of sensor-based devices capable of detecting eating and drinking and to evaluate whether recent advancements have improved their feasibility for use in real-world nutrition applications.

**Methods:**

A scoping review was conducted using the Preferred Reporting Items for Systematic reviews and Meta-Analyses for Scoping Reviews framework. Studies published between January 2022 and September 2025 that evaluated the performance of sensor-based devices in identifying food and/or beverage intake were included. Devices were evaluated against 6 feasibility criteria to assess real-world applicability: ≥80% accuracy, freedom in food and beverage selection; social acceptability and comfort; long battery life; real-time detection; and ability to detect both eating and drinking.

**Results:**

Fifty studies (52 devices) were included: 19 wrist-worn, 8 neck-worn, 7 ear-worn, 7 glasses-type, 6 in the “other” category, and 5 multiposition devices. None met all 6 feasibility criteria. The most common unmet criterion was adequate battery life (*n* = 43), followed by real-time processing (*n* = 37), variety of foods or behaviors in testing (*n* = 31), detection of both eating and drinking (*n* = 31), social acceptability and comfort (*n* = 15), and accuracy (*n* = 10).

**Conclusions:**

Although no sensor-based devices met all criteria for real-world feasibility, recent advancements suggest meaningful progress in areas of social acceptability and computational efficiency. These improvements signal a shift toward more practical, user-friendly designs that may soon be capable of supporting automated dietary assessment and individualized nutrition care.

**Statement of Significance**

Rapid advancements in sensor-based detection of eating indicate growing potential for integration into real-world nutrition care. This scoping review evaluates whether recently developed devices have overcome past limitations in feasibility, identifying those most ready for field-based dietetic implementation.

## Introduction

The impact of dietary habits on health is well-documented [[1], [2], [3], [4]], with the WHO labeling unhealthy diets as one of the 4 risk-related behaviors common to noncommunicable diseases [1]. In fact, diet-related deaths accounted for nearly 11 million deaths in 2017 [5]. Despite this, our ability to accurately assess dietary intake to study diet–disease relationships and establish causation, as well as to assess food intake in nutrition interventions, remains a challenge. For years, traditional dietary assessment methods such as food diaries, diet histories, and 24-h recalls and their digital counterparts have remained the cornerstone to nutrition assessment [6]. Nutrition assessment is one of the 4 integral steps in the Nutrition Care Process, a framework that guides the Medical Nutrition Therapy that dietitians provide, alongside nutrition diagnosis, nutrition intervention, and nutrition monitoring/evaluation [7]. However, these traditional dietary assessment methods rely on individuals to self-report their intake, requiring them to either remember to initiate recording or accurately recall what they consumed, making the process prone to error and burdensome for both the respondent and dietitian [8].

Since the early 2010s, there has been rapidly growing interest in automating the process of eating detection, such as via machine learning and sensor-based approaches [9,10]. These technologies have the potential to advance the Nutrition Care Process by improving the accuracy of dietary assessment, reducing its burden, and improving the delivery of dietary interventions. For example, the real-time detection of eating occasions could be used to either trigger event-contingent ecological momentary assessment (EMA), prompting individuals to log or capture an image of their intake [11,12], or automatically switch on wearable cameras to record food consumption [13,14]. This, potentially combined with automated image analysis [[15], [16], [17]], could eliminate or minimize recall bias and reduce the time burden on both respondents, when using image-based methods, and dietitians, who would otherwise spend a substantial amount of time recording dietary intake during consultations [18]. Additionally, sensor-based data can provide precise insights into temporal eating patterns, such as meal timing and duration [19]. In the context of dietary interventions, continuous intake detection could support the delivery of just-in-time (JIT) feedback [20], an approach designed to provide the “right support” at the “right time” [21]. This strategy may help prevent dietary lapses and improve adherence to predetermined dietary goals [22,23].

Beyond the Nutrition Care Process, these features also highlight the potential for wearable sensors to be central to interventional personalized nutrition and N-of-1 trials, study designs in which a single individual serves as their own control by undergoing repeated, often randomized, testing of different interventions, or observational N-of-1 trials, in which multiple measures of behavior or physiology are recorded over time without an intervention [24]. In both cases, there is an emphasis on continuous, real-time data collection [25], which has conventionally been gathered using self-reported methods such as signal-contingent EMA [26] and questionnaires [27], or technology sensor-based methods such as wearable devices but primarily for physical activity and sleep [28]. Replacing these self-reported methods with the continuous, context-aware data provided by wearable sensors and event-contingent EMA or images can not only reduce burden but also provide more granular insights on the personal and contextual factors that influence food intake decisions [29]. This approach, still relatively novel in the context of Medical Nutrition Therapy, is particularly valuable for capturing the substantial individual variability in dietary responses often obscured in traditional randomized controlled trials focused on population-level effects [30,31].

Despite the many benefits sensor-based devices can offer to the Nutrition Care Process and emerging research methodologies, developing, deploying, and evaluating these systems remains a challenge [9]. A scoping review conducted by the authors 4 y ago found that sensor-based devices for detecting eating and drinking were not yet suitable for dietetic practice [10]. Limitations included insufficient accuracy, the inability to detect both eating and drinking, lack of testing in free-living settings, wearer discomfort, primitive prototypes, and inadequate battery life. However, given the rapid development of this field, more recent research may have led to new advancements. Therefore, the aim of the present scoping review is to provide an update to the previous review and assess whether any new devices have been developed to meet the feasibility requirements for use in real-world nutrition applications.

## Methods

### Protocol and registration

No protocol was developed or registered for this current scoping review. However, the review was informed by a previous review conducted by the authors [10], for which a protocol was prospectively registered using the Open Science Framework on 11 January, 2022 (https://doi.org/10.17605/OSF.IO/EYH5A).

### Eligibility criteria

The methodology of this scoping review has previously been described elsewhere [10]. In brief, the review followed the Joanna Briggs Institute and PRISMA for Scoping Reviews protocols. Articles were eligible if they were published in English between January 2022 and 5 September 2025 and described sensor-based devices that passively detect eating and/or drinking in real time. This time frame was selected as it followed on from the prior scoping review conducted between January 2016 and December 2021. Only the most recent publication from each research group was included. Exclusions applied to animal studies; devices requiring user input; or those used solely for dietary interventions, enteral/parenteral feeding, or other daily activities not involving ingestion. Web- and mobile application-based tools were excluded, as were papers lacking classifier performance evaluation or hardware design details (Table 1).

**TABLE 1:** Inclusion and exclusion criteria of the scoping review

Selection criteria	Inclusion criteria	Exclusion criteria
Population	• Adult participants aged ≥18 y • Participants ≤17 y if the device can potentially be used in adults	• Animal studies
Concept	• Devices that identified and recorded, or had the capacity to identify and record, the start time of an eating and/or drinking occasion over multiple days in real time • Passive, sensor-based devices that did not require user input • The most recent paper published by a research group about a particular device	• Instruments that measured usual, mean, or overall intake but did not identify and record the start time of eating and/or drinking • Devices used solely for administering dietary interventions • Devices that measured enteral or parenteral feeding • Devices that were displaced or discontinued at the time of the search • Devices requiring user input • Web-, mobile application-, or paper-based dietary assessment tools • Devices that measured other activities of daily living apart from eating or drinking
Context	• Papers published between January 2022 and 5 September, 2025 inclusive and in English • Case studies, conference proceedings, dissertations, and opinion papers in peer-reviewed and gray literature • No geographic limitations • Studies conducted in laboratory, semi-controlled, or free-living settings, provided the device was feasible for free-living use regarding user/social acceptability and comfort • Any study or review evaluating device performance	• All papers published before 2022 • Papers published in languages other than English • Papers that did not evaluate device performance • Papers solely describing data-processing pipelines or algorithms without hardware design descriptions

### Information sources and search

The search strategy was applied in 7 electronic databases: ACM digital library, CINAHL (EBSCO), EMBASE (Ovid) (EMBASE, RRID:SCR_001650), IEEE Xplore (IEEE), PubMed, Scopus (Elsevier), and Web of Science (Clarivate Analytics). Sources of gray literature searched included: Google, Trove, MedNar, and official websites of international and government organizations. The complete search strategy for Ovid MEDLINE can be found in the Supplementary Material. The final search results were exported into EndNote 21.2 (EndNote, RRID:SCR_014001) and duplicates were removed.

### Screening and selection of evidence

The titles and abstracts of all studies were reviewed against the eligibility criteria. If the title and/or abstract mentioned a sensor-based device that detected eating or drinking, the study was included in the initial screening stage to be assessed in the full-text screening stage.

Studies were further excluded during the full-text screening stage if they did not evaluate eating or drinking detection performance, were not feasible for use in free-living settings, were not evaluating a physical device or had no experimental section, or if a more recent study by the same research group superseded earlier work on the same device within the review timeframe (2022–2025). Screening and selection of evidence were completed by 1 reviewer (LW).

### Data charting process and data items

A data extraction table developed during the previous scoping review was used. In this table, the devices were categorized according to device placement. For each device, the following data items were included:

1) The type(s) of sensors used, and the number of each sensor included in the device.
2) The type(s) of intake (for example, food and beverages) and eating proxy or gesture (for example, hand-to-mouth motions, chewing, swallowing, etc.) measured.
3) Ground truth method, evaluation metric(s), and performance of sensors.
4) Advantages and disadvantages of each study, including aspects related to study design and the feasibility of the device for supporting dietary assessment in real-world settings, evaluated against the 6 feasibility criteria described below.
5) Experiment details including the setting, duration, number of food and/or beverage types tested, and number of participants.
6) The data-processing pipeline including the classification algorithm used, sampling rate, and the number of features.

### Synthesis of results

Devices that passed full-text screening were further evaluated against 6 feasibility criteria to identify those suitable for real-world dietary assessment. These feasibility criteria were selected based on the authors’ prior scoping review [10] and involved a combination of expert opinion from senior registered dietitians (MA-F, AR) as well as previous literature. The 6 criteria, along with their justifications, are as follows:

1) A mean performance accuracy or F1-score of ≥80% in detecting eating and/or drinking must be achieved. This cut-off was selected based on the performance of a Bite Counter device used in dietitian-led trials [32,33], which achieved an F1-score of 82% in free-living settings [34].
2) The device must have been tested in free-living settings or laboratory settings where participants were free to choose their own foods and activities to reflect real-world eating behaviors.
3) The device had to be discreet, socially acceptable, and comfortable for the user to wear for long durations to promote user acceptability, sustained use, and potential for widescale adoption. Social acceptability was determined based on user feedback where available, as well as the appearance and description of the device hardware, at the researchers’ discretion. Given the emerging nature of this field, devices were not required to match the design quality or aesthetic of commercial, off-the-shelf wearables. However, devices that appeared large, obtrusive, or difficult to disguise as everyday accessories were considered socially unacceptable.
4) A battery life of 12 h or more on a single charge was required to ensure that around 1 waking day could be captured without interruption for charging.
5) The data-processing pipeline and algorithm needed to classify data in real time and within 5 min to account for brief snacking events and enable timely detection to support potential future applications where wearable sensors could prompt users to record their intake upon detection.
6) The device must have the ability to detect both eating and drinking gestures as beverages contribute ≤1 quarter of total daily energy intake [35,36].

## Results

### Study selection

**FIGURE 1:**
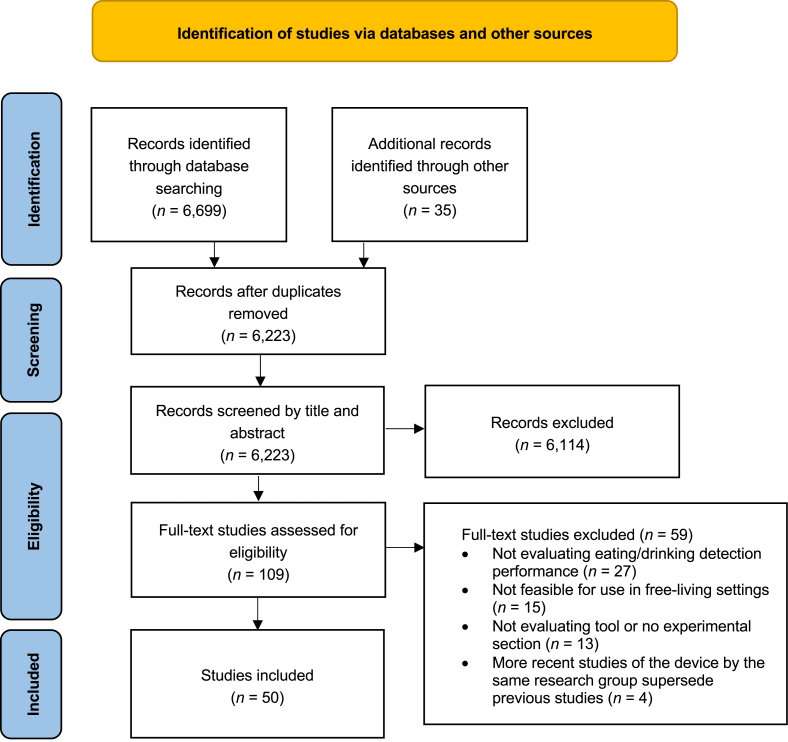
Flow chart of included studies in the scoping review.

As shown in Figure 1, the search identified 6699 records. After deduplication, 6223 studies were screened by title and abstract, of which 6114 were excluded. The remaining 109 records underwent full-text review. Fifty studies met the inclusion criteria and were included in the scoping review. The reasons for full-text exclusion are provided in Figure 1, which include: *1*) an absence of evaluation of the device’s performance in detecting eating or drinking, *2*) the device’s unsuitability for free-living settings, *3*) a lack of details regarding the experimental setup or hardware, with the study focused solely on the data-processing pipeline, and *4*) the supersession of the study by a more recent paper of the same device by the same research team.

### Study characteristics

The 50 included studies were developmental and evaluation studies describing 52 devices, of which 19 were wrist-worn [[37], [38], [39], [40], [41], [42], [43], [44], [45], [46], [47], [48], [49], [50], [51], [52], [53], [54], [55], 8[37–55], 8 were neck-worn [[56], [57], [58], [59], [60], [61], [62], [63], 7[56–63], 7 were ear-worn [[64], [65], [66], [67], [68], [69], [70], 7[64–70], 7 were glasses-type devices [[71], [72], [73], [74], [75], [76], [77], 6[71–77], 6 were categorized as “other” [52,[78], [79], [80], [81]], and 5 were multiposition devices [[82], [83], [84], [85], [86]]. Most devices were assessed for their performance in detecting eating only (*n* = 25), 21 were assessed for their performance in detecting both eating and drinking, and 6 were assessed for drinking only. Seven of the devices [54,65,[71], [72], [73], [74],^76^,^78^] were updated versions of earlier prototypes developed by the same research groups and reported in our prior scoping review [[87], [88], [89], [90], [91], [92]].

No devices identified in this scoping review met all 6 feasibility criteria. Three devices met 5 of the 6 criteria; 1 was designed as a pair of glasses that monitored chewing [75], [2] were designed as a neck-worn, activity-oriented wearable camera [60,62]. The unmet criteria were the ability to detect both eating and drinking [62,75], and a socially acceptable design, specifically the size of the device prototype [60] (Table 2). Ongoing advancements in miniaturization and personalization are likely to improve the device’s acceptability [60]. Twelve devices fulfilled 4 of the 6 criteria [43,44,[46], [47], [48],^53^,^55^,^56^,^71^,[76], [77], [78]]. Insufficient or lack of reporting on battery life was the most common unmet feasibility criterion (*n* = 43), particularly among multiposition devices. This was followed by an absence of testing for the device’s capacity for real-time processing (*n* = 37), the use of a limited number of foods and/or behaviors to test device models (*n* = 31), an inability to detect both eating and drinking behaviors (*n* = 31), socially unacceptable or uncomfortable device designs (*n* = 15), and an eating or drinking episode detection accuracy of <80% (*n* = 10).

**TABLE 2:** An overview of all identified devices from the updated scoping review search compared with the 5 6 feasibility criteria used to determine suitability for use by dietitians in real-world settings

Reference	Accuracy^1^	Real-world applicability^2^	Social acceptability^3^	Battery life^4^	Real-time processing^5^	Both eating and drinking^6^
Wrist-worn devices (*n* = 19)						
Bhattacharya 2022 [37]	✗	✓	✓	✗	✗	✓
Liang 2022 [38]	✗	✗	✓	—	✗	✓
Diamantidou 2022 [39]	✓	✗	✓	—	✗	✓
Mekruksavanich 2022 [40]	✓	✗	✓	✗	✗	✓
Mekruksavanich 2022 [41]	✓	✓	✓	—	✗	✗
Sharma 2022 [42]	✗	✓	✓	—	✓	✗
Zhang 2022 [43]	✓	✓	✓	—	✗	✓
Zhang 2022 [44]	✓	✓	✓	—	✗	✓
Al Jlailaty 2023 [45]	✓	✗	✓	—	✗	✓
Wang 2023 [46]	✓	✓	✓	—	✗	✓
Wei 2023 [47]	✓	✗	✓	✓	✓	✗
Dénes-Fazakas 2024 [48]	✓	✓	✓	—	✓	✗
Hsieh 2024 [49]	✓	✗	✓	—	✗	✗
Liu 2024 [50]	✓	✗	✗	✓	✗	✓
Martinez 2024 [51]	✓	✗	✓	—	✓	✗
Mevissen 2024 [52]	✓	✗	✓	—	✗	✗
Wang 2024 [53]	✓	✓	✓	✓	✗	✗
Xuan 2025 [54]	✗	✓	✓	—	✗	✗
Hou 2025 [55]	✗	✓	✓	—	✓	✓
Total "✓"	14	10	18	3	5	10
Neck-worn devices (*n* = 8)						
Khan 2022 [56]	✓	✗	✓	✗	✓	✓
Chen 2023 [57]	✓	✗	—	—	✗	✓
Shahi 2023 [58]	✗	✓	✗	—	✗	✗
Ismail 2024 [59]	✓	✗	✗	—	✗	✗
Shahi 2024 [60]	✓	✓	✗	✓	✓	✓
Ghosh 2025 [61]	✓	✗	✗	—	✗	✓
Fernandes 2024 [62]	✓	✓	✓	✓	✓	✗
Chen 2025 [63]	✓	✗	✗	—	✓	✗
Total "✓"	7	3	2	2	4	4
Ear-worn devices (*n* = 7)						
Morshed 2022 [64]	✓	✓	✓	—	✗	✗
Odame 2022 [65]	✓	✗	✗	✓	✓	✗
Hossain 2023 [66]	✓	✓	✗	—	✗	✗
Tan 2023 [67]	✓	✗	✓	—	✗	✗
Ketmalasiri 2024 [68]	✓	✗	✓	—	✗	✗
Mekruksavanich 2024 [69]	✓	✗	✓	—	✗	✗
Khurana 2025 [70]	✓	✗	✓	—	✗	✓
Total “✓”	7	2	5	1	2	1
Glasses devices (*n* = 7)						
Bedri 2022 [71]	—	✓	✓	✓	✓	✗
Papapanagiotou 2022 [72]	✓	✗	✓	—	✗	✗
Saphala 2022 [73]	✓	✓	✓	✗	✗	✗
Selamat 2022 [74]	✓	✗	✓	—	✗	✗
Shin 2022 [75]	✓	✓	✓	✓	✓	✗
Ghosh 2024 [76]	✓	✓	✓	—	✗	✓
Stankoski 2024 [77]	✓	✓	✓	—	✓	✗
Total “✓”	6	5	7	2	3	1
Other devices (*n* = 6)						
Bi 2022 [78]	✓	✓	✓	✓	✗	✗
Liu 2022 [79]	✗	✗	✗	—	✗	✗
Kim 2023 [80]	✓	✗	✓	—	✓	✗
Mevissen 2024 [52]	✓	✗	✗	—	✗	✗
Mevissen 2024 [52]	✗	✗	✓	—	✗	✗
Scattolini 2024 [81]	✓	✗	✓	—	✗	✓
Total “✓”	4	1	4	1	1	1
Multiposition devices (*n* = 5)						
Woodward 2022 [82]	✓	✗	✗	—	✗	✓
Yeh 2022 [83]	✓	✗	✗	—	✓	✗
Karmakar 2023 [84]	✗	✗	✗	—	✗	✓
Wang 2024 [85]	✓	✗	✓	—	—	✓
Mekruksavanich 2025 [86]	✓	✗	✗	—	✗	✓
Total “✓”	4	0	1	0	1	4

“✓” indicates that the criterion has been met; “✗” indicates that the criterion has not been met; and “—” indicates that the criterion was not reported by the study and could not be inferred by the authors.

1 A mean accuracy or F1-score of ≥80% in detecting individual eating proxies, eating bouts, or entire eating episodes.

2 Device testing occurred in free-living, semi-free-living, or laboratory settings where participants were free to choose their own foods and activities.

3 A device that was discreet, socially acceptable, and comfortable for the user to wear for long durations determined by user feedback or the appearance and description of the device hardware.

4 The study reported a battery life on a single charge of >12 h or 1 waking day.

5 Sensor data were classified in real time as either eating or not eating within 5 min.

6 Devices that could detect, but not necessarily distinguish between, eating and drinking events.

### Results by device type

Table 3 and the subsections below summarize the sensor-based devices by device type, outlining each device category’s strengths and limitations.

**TABLE 3:** Methods and performance of sensor-based devices identified in the updated scoping review search that detect food and/or beverage intake (*n* = 52)

Reference	Sensors^1^	Intake type^2^	Ground truth/results^3^	Advantages^4^	Disadvantages^4^	Setting^5^	Processing^6^
Wrist-worn devices (*n* = 19)							
[37]	Accelerometer (1) Gyroscope (1) Microphone (1)	Eating snacks Drinking HTM motions Contextual sounds close to the body	Ground truth: Video recording Overall macro and weighted F1-scores: 30% and 55.8% Eating snacks F1-score: 65.9% Drinking F1-score: 50%	Commercial, unmodified Fossil Gen 4 smartwatch used. Data made available as a public dataset.	Ground truth was 25 s video for every min of data. Video labeled as whichever activity occupied most of its duration. Eating snacks and drinking classified of 21 other nonconsumption activities. 3.5 h battery life. Performance impacted by background audio and multitasking.	Free-living 2 sessions per subject. Each session lasted the duration of device battery life (∼3.5 h) 5 subjects	Accelerometer and gyroscope: 50 Hz Microphone: 22.05 kHz 10 s window with 50% overlap 48 frame-level features e-CNN14 model with concatenation
[38]	Accelerometer (1) Gyroscope (1) Microphone (1)	Eating snacks Drinking HTM motions Contextual sounds close to the body	Ground truth: Video recording F1-score: 74.4%	Commercial, unmodified Fossil Gen 4 smartwatch used. New teacher-student model that addresses privacy issues from audio data.	Ground truth was a video call conducted by researchers. Teacher-student model increases computational burden and time cost.	Semifree-living 23 activities of daily living 2 × 30 s each 15 subjects	Accelerometer and gyroscope: 50 Hz Microphone: 22.05 kHz 10 s window with 50% overlap Inertial: DeepConvLSTM Audio: Audio CNN
[39]	Accelerometer (1) Gyroscope (1)	Eating Drinking HTM motions	Ground truth: manual annotation by subjects	Commercial Samsung Galaxy Watch 3 used.	Classification was only between 4 activities performed nonsimultaneously, in-lab.	In-lab 4 activities (eating, drinking, smoking, idle) 1 h each 12 subjects	50 Hz 16 human-crafted features 3 s window with 50% overlap
[40]	Accelerometer (1) Gyroscope (1)	Eating Drinking HTM motions	Ground truth not specified Accuracy: 97.4%	Commercial LG G Watch running Android Wear 1.5 used. Large, publicly available dataset (WISDM) used	Small range of foods and beverages used—soup, chips, pasta, sandwich, drinking from a cup	In-lab 18 activities (5 eating/drinking activities) 3 min each 51 subjects	20 Hz Stacked LSTM step size 20
[41]	Accelerometer (1) Gyroscope (1)	Eating (not further defined) HTM motions	Ground truth: Mark start/end of each meal F1-score: 91.81%	Commercial Mobvoi TicWatch S used. Model able to differentiate fork, knife, spoon, combo of above, and hand	Drinking occasions not evaluated	Free-living Total 481 h 10 min 12 subjects	100 Hz ResNet-SE
[42]	Accelerometer (1) Gyroscope (1)	Eating (not further defined) HTM motions Other wrist motion for context	Ground truth: Mark start/end of each meal TPR: 89% FP/TP: 1.7 F1-score: 48%	Commercial Shimmer3 used. Large, publicly available dataset (Clemson All-day) used. Start of meals detected 1.5 min prior — potential for just-in-time intervention	Analysis of longer windows results in missing small snacks	Free-living 1 day 351 subjects	15 Hz Window size 6 min CNN then LSTM
[44]	Accelerometer (1) Gyroscope (1)	Eating Drinking HTM motions	Ground truth: Mark start/end of each meal Weighted F1-score: 0.870	Commercial Apple Watch Series 4 used.	Eating periods <3 min excluded, so small snacks may be missed. False positive rates higher between 06:00 and 07:00 and at 21:00, indicating more eating-like HTM then	Free-living 22 d 17 subjects	50 Hz 20 features Deep CNN
[43]	Accelerometer (1) Gyroscope (1)	Eating Drinking HTM motions	Ground truth: Video recording F1-score: 73.7% and 83.8% for 3 s and 15 min time resolutions	Device used was not commercially available, but same sensors can easily be found on commodity smartwatches. 3 s windows can capture small snacks.	Head-to-food movements may not be captured, e.g. when eating burgers or sandwiches.	Free-living 2 h/1 meal 9 subjects	30 Hz downsampled to 8 Hz Deep neural network
[45]	Accelerometer Gyroscope (1) each for FIC, Clemson cafeteria (2) each for OREBA	Eating Drinking HTM motions	Ground truth: video recording F1-score: 92%, 94%, 95%, 85% for OREBA-SHA, OREBA-DIS, FIC, Clemson cafeteria datasets	Commercial Movisens Move 3+ used for OREBA. Commercial Microsoft Band 2 and Sony Smartwatch 2 used for FIC. Large, publicly available datasets used	All datasets used were in-lab	In-lab 1 meal OREBA-DIS: 100 OREBA-SHA: 102 FIC: 12 Clemson cafeteria: 271 subjects	OREBA: 64 Hz FIC: 100 Hz Clemson cafeteria: 15 Hz SVM
[46]	Accelerometer (1) Gyroscope (1)	Eating Drinking HTM motions	Ground truth: video recording F1-score: 82.6% and 89.3% for eating and drinking	Commercial Shimmer3 used. Potential for real-time processing as sampling rate of 16 Hz may reduce power consumption and data storage	Not a full day of data collection. Head-to-food movements may not be captured, e.g. when eating burgers or sandwiches	Free-living 2 main meals, 1 snack, ∼5 h 12 subjects	64 Hz downsampled to 16 Hz MS-TCN
[47]	Accelerometer (1) Gyroscope (1)	Eating (not further defined) HTM motions	Ground truth: video recording F1-score: 81.6%	Large, publicly available dataset (Clemson cafeteria) used. 25 h battery life when tested on commercial Fossil Gen 5 Julianna HR smartwatch and Google Pixel 5 for real time, continuous detection. 15.97 ms inference time for every 2.2 s of data	Clemson cafeteria dataset is in-lab. Inference time more applicable for short duration eating events	In-lab 1 meal 264 participants	15 Hz O-MCC template-based algorithm
[48]	Accelerometer (1) Gyroscope (1) Magnetometer (1)	Eating only HTM motions	Ground truth: Mark start/end of each meal to nearest second F1-score: 98.9%	Commercial Shimmer3 used. Model could be run on a phone as app. Model’s average prediction latency was 5.5 s. Large, publicly available dataset (Clemson All-day) used	Actigraph GT9X no longer commercially available. Utilizes personalized model, which would be less applicable to public	Free-living 1 d 351 participants	15 Hz 12 features LSTM
[49]	Accelerometer (1) Gyroscope (1)	Drinking only HTM motions	Ground truth: video recording F1-score: 92.89%	Commercial Opal sensor used	Tested on one smart cup only. Other drinking styles, e.g. drinking from straw, not considered	In-lab 84 drinking events pp 12 subjects	128 Hz 8 features SVM
[50]	Electrodes (2) measuring bioimpedance	Eating Drinking Food type	Ground truth not specified F1-score: 86.4% and 64.2% for detecting intake and classifying 7 food types	Compact lithium battery (500 mAh) could power system for ∼1 d. Eating with knife/fork most easily distinguished as both hands used, forming a circuit. Clear distinction between eating and drinking.	Performance impacted by placement of electrodes, width between knife and fork, participants’ height and weight. If duration between cutting and eating is very short (e.g. fast eaters or soft foods), both actions fit into one window which confuses model during training. When classifying food types, there is confusion between foods with similar texture or water content, e.g. tomato vs. cucumber, sausage vs. meat	In-lab 4 meals Knife/fork — steak, sausage, cucumber, tomato, banana Hand — bread, apple, orange Metal cup and straw — natural or vitamin water 10 subjects	60 kHz sinus signal without DC bias added via electrodes Output voltage is 50 mV peak to peak AFE output data rate 20 Hz Window size 30 (1.5 s) Features from Magnitude and Phase components
[51]	Accelerometer (1) Gyroscope (1)	Drinking only HTM motions	Ground truth: Timing of fluid intake recorded. F1-score: 100%	Commercial Witmotion Bluetooth 2.0 BWT901CL model used. Onset of action defined 1 s before start time	In-lab study with limited activities—standing or sitting while drinking liquids, touching hair/adjusting glasses	In-lab 10 subjects	50 Hz
[52]	Accelerometer (1) Gyroscope (1)	3 foods HTM motions	Ground truth: video recording F1-score: 82%	Commercial Huawei Watch 2 used. Potential for beverages to be detected in the future	In-lab study with limited activities—scratch back of head, random arm movement—and foods—apple, yogurt, croissant	In-lab 3 food types 6 subjects	83 Hz 1 s window 13 features SVM
[53]	Accelerometer (2) Gyroscope (2)	Eating HTM motions	Ground truth: video recording F1-score: 92.8% and 89.7% for FD-I and II	Commercial Shimmer3 used. Also able to determine eating speed; potential for just-in-time intervention	In FD-I, ground truth captured at location A and B, but not during transit (although researcher observation present). In FD-II, ground truth captured at main meals only. Eating periods <3 min excluded, so small snacks may be missed	FD-I: near-free-living 2 locations 34 subjects FD-II: free-living Main meals 27 subjects	64 Hz downsampled to 16 Hz TCN-MHA k=0.1
[54, 62]	Accelerometer (1) Gyroscope (1) Magnetometer (1)	Meals Snacks HTM motions	Ground truth: mark start/end of each meal/snack Weighted accuracy: 77.9%	Commercial Shimmer3 used. Potential for beverages to be detected in the future as utilizes HTM	Detected eating occasions <1 min excluded, so small snacks may be missed. Utilizes personalized model, which would be less applicable to public	Free-living 9–21 d 9 subjects	15 Hz 6 min sliding window with stride 15 s Window-based CNN Model trained on Clemson All-day dataset then incrementally trained on target individual using transfer learning
[55, 63]	Accelerometer (1) Gyroscope (1) Microphone (1)	Eating Drinking HTM motions	Ground truth: video recording 79.75% temporal Intersection over Union for eating time detection 86.0% accuracy for food type classification	Commercial Samsung Galaxy Watch 5 and Google Pixel Watch 2 used. Different utensils used (e.g. fork, spoon, chopsticks, cup, bottle) and able to classify by food type (staple, hard/crispy, soft, fruits/veg, beverages)	Performance impacted by level of background noise. Potential privacy concerns with audio. Each eating moment required ≥4 gestures per min and last for a minimum duration of 3 min, which may miss small snacks	In-lab Total >400 eating sessions (10 min each with eating and noneating activities) 18 subjects	41 kHz for microphone Inertial: 3 s windows Sequence modeling: Bi-GRU/GRU Conv-TasNet: for acoustic denoising Classifiers: dense layers + softmax
Neck-worn devices (*n* = 8)							
[56]	Condenser electret microphone (1)	3 foods Water Chewing Swallowing	Ground truth: Annotation of audio F-score: 81.25% for classifying between drinking, chewing, and nonintake	Device constructed from components that are commercially available. Data transmitted to a smartphone for real-time classification. Able to distinguish liquid and dry swallows	10 h battery life may not be suited for individuals with prolonged eating windows. Performance relatively lower for drinking detection	In-lab 5 nonintake activities — speech, cough, laugh, breathe, dry swallow 20 subjects	76 features Random forest
[57]	Microphone (1)	Cookies Water Swallowing sounds	Ground truth not specified F1-score: 95%	Coffee shop noise added during classification to simulate free-living. Swallowing saliva also captured during in-lab session	More details about the device, including type of microphone, and ground truth method could be described. Unable to distinguish between food, beverage, and saliva swallow. Performance impacted by head movement	In-lab 10 activities 7 subjects	44.1 kHz 4 features 0.2 s window size Template matching
[58]	RGB camera (1) Low-resolution IR sensor (1)	Eating only HTM motions Head-to-food motions	Ground truth: video recording F1-score: 70%	Able to capture head-to-food movements (e.g. eating burger) not usually captured in HTM proxies due to camera positioned up toward face. Commercial WildCam used. Night vision capability. Able to detect social context.	No beverages classified. Impacted by confounding HTM gestures	Free-living 3 d 10 subjects	5 Hz 2 stream CNN
[59]	Microphone (1)	Drinking only Swallowing	Ground truth not specified F1-score: 87.5%	Able to distinguish liquid swallow from saliva swallow. Commercial RØDE SmartLav+ Smartphone Lavalier Microphone used	Beverages only and conducted under stringent in-lab conditions	In-lab 10 activities 11 subjects	44.1 kHz 3 features 1.2 s window size SVC or LDA
[60]	Thermal sensor (1) Camera (1)	Eating Drinking HTM Object-in-hand	Ground truth: video recording F1-score: 89%	Eating episodes can be detected using 10 gestures or within first 1.5 min. Combining HTM and object-in-hand improves F1-score by ≥34% as confounding HTM distinguished. Supports timely ML model execution in real time, on device	Very short eating episodes <1.5 min, e.g. one piece of chocolate, may be missed	Free-living 1 d for 8 subjects, 7 d for 14 subjects, 14 d for 14 subjects 36 subjects total	5 frames per s for both sensors DBSCAN
[61]	Antenna operating at 2.4 GHz (2) measuring disturbances in nearfield	Eating Drinking Chewing Swallowing	Ground truth not specified Accuracy: 98.34% From confusion matrix, 95.8% and 97.9% for eating and drinking	High accuracy when distinguishing eating and drinking from coughing, deep breathing, and idleness	Collection limited to 10 s to prevent fatigue and discomfort. 5 activities classified in-lab. Types of foods and beverages not specified. Saliva swallow not assessed	In-lab 5 activities 8 subjects	100 Hz 2.5 s window with 50% overlap DCN-Net for smoothed GWT
[62]	Accelerometer (1) Thermal sensor (1) RGB camera (1)	Eating HTM motions	Ground truth: subjects mark start/end of each episode via app + video recording Eating detection Offline models RGB data F1-score: 89.7% Thermal data F1-score: 85.6% On-device model Thermal data F1-score: 59.5%	Privacy-preserving features: activity-oriented camera; RGB recording only when wearing and HTM detected via accelerometer and thermal sensor; blurred background. Long battery life >12 h. 53.33% of participants were neutral re social acceptability; 47% were comfortable. Potential for drinking detection as uses HTM	Lower performance during real-time classification due to use of thermal data to minimize computational demands	Free-living Total 768 h of data collected (mean daily wear time 7.2 h) 13 subjects	5 Hz Offline analysis: TimeSformer On device: quantized 2-layer fully connected neural networks
[63]	RGB camera (1)	Drinking HTM motions	Ground truth: video recording F1 score: 0.879 Precision: 0.844 Recall: 0.918	Short latency of 0.29 and 0.07 s for start and end of drinking episodes. Eating data also collected as a “nondrinking activity” so potential for detection in future studies.	Bulky device design. Potential privacy concerns. Detection may be impacted in low-light environments	In-lab Drinking with different utensils (total 9 h 55 min) Nondrinking activities (total 8 h 45 min) 16 subjects	CNN—YOLOv8 pretrained on COCO dataset
Ear-worn devices (*n* = 7)							
[64]	Accelerometer (1) Gyroscope (1)	19 foods Chewing	Ground truth: Video recording F1-score: 58% F1-score: ∼90% when trained on 2 min of participant’s own data	Commercial Nokia eSense earbuds used. Participants could choose any snack they preferred. Data transferred to connected Google Pixel 5 smartphone in real time. Researchers distracted participants during session to prevent prolonged chewing	May falsely classify gum as chewing is a proxy. Soft foods, e.g. yogurt, may be missed and eating while walking may impact performance	In-lab 19 food types 18 subjects	25 Hz downsampled to 10 Hz 36 features Random forest
[65]	Contact microphone (1)	6 foods Chewing	Ground truth not specified F1-score: 94%	24-s resolution in identifying chewing events	May falsely classify gum as chewing is a proxy. Soft foods, e.g. yogurt, may be missed. Unable to detect beverages	In-lab 6 foods 20 subjects	500 Hz ANN
[66]	Pressure sensor (1) Accelerometer (1)	Eating only Chewing	Ground truth: wearable camera images every 10 s F1-score: 0.887	Custom molded earbud for comfort. Potential to trigger wearable camera to automatically capture intake. Potential to capture eating speed as model counts chews. Short eating episodes can be detected. Device can record sensor data and images for 20.5 h.	Not commercial device. May falsely classify gum as chewing is a proxy. Soft foods, e.g. yogurt, may be missed. Unable to detect beverages. Not real-time processing	Free-living 1 day 3 subjects	128 Hz for both sensors 8 features 5 s epoch SVM
[67]	Speaker-only headphone (1) measuring change in ear canal pressure	Eating only Chewing	Ground truth not specified MAE: 0.865 RMSE: 1.26 Average error rate: 0.11 F1-score: 88.4% based on training data	Commercial headphone can be used	Headphones not suitable for social eating. More error when detecting crispy foods. Performance varies from person to person. Gum considered food. No beverages classified	In-lab 13 foods, including gum 5 subjects	24 features Sliding window 7 s RBF kernel SVM
[68]	Accelerometer (1) Gyroscope (1) Earbud in left ear	Eating only Chewing	Ground truth for eating episode: video recording Ground truth for individual chews: subjects pressed spacebar for each chew	Able to capture eating speed as model counts chews. Commercial Nokia Bell Labs eSense used. Unobtrusive device	Chew count performs better for slower chews and more chewy foods (bread). Chew count had more errors for soft foods (mango). Potential discomfort with long term wear. Simultaneous activities not assessed	In-lab 5 foods 8 noneating activities 8 subjects	60 Hz 96 features (18 time domain, 78 frequency domain) Random forest
[69]	Accelerometer (2) Gyroscope (2) PPG (2) for vital signs	Eating only Chewing	Ground truth not specified Accuracy 98.18% F1-score 98.29%	Small, discreet device	More details about the device and experimental setup could be described. May falsely classify gum as chewing is a proxy. Soft foods, e.g. yogurt, may be missed. Potential discomfort with long term wear	In-lab 16 head and face movement, including chewing 30 subjects	2 s window with 50% overlap CNN-BiLSTM
[70]	Microphone (1)	Eating Drinking Chewing Swallowing	Ground truth not specified Accuracy: 0.9643 Precision: 0.954	May be implemented on commercial devices. Likely less impacted by environmental noise compared with microphones positioned at other locations	Headphones not suitable for social eating. Gum, soft foods (e.g. yogurt), or dry swallows may be misclassified	In-lab 12 meals of different textures	Hybrid BiLSTM HMM
Glasses-type devices (*n* = 7)							
[71]	Proximity sensor (1) for HTM Gyroscope (1) for chewing IMU (1) as reference	Eating only Chewing HTM motions	Ground truth: subjects click “no” on app when not eating, measuring false positives. False negatives not measured. Subjects received 0-11 false positives per day	Sensors designed to be attached to any pair of eyeglasses. FitNibble sends notifications to remind users to log every time it detects eating (event-contingent ecological momentary assessment). Battery life not explicitly mentioned, but assuming 9 d as 9-day in-field study	More suitable for people already wearing glasses. Accuracy heavily reliant on proper placement of sensor. Notification sent when 5 consecutive responses infer eating, potentially missing very small snacks, e.g. chocolate.	Free-living 7 d of data collection 13 subjects	10 Hz 7 features Unoptimized DNN
[72]	Microphone (2)	5 foods Chewing	Ground truth: mark start/end of each food. Researchers annotated each chew F1-score: 96%	Commercial Razer Anzu smart glasses used	May falsely classify gum as chewing is a proxy. Soft foods, e.g. yogurt, may be missed. Eating while walking may impact performance	In-lab 20 min of eating, 10 min of other activity 5 subjects	16 kHz “Small set of carefully selected features” Binary SVM classifier
[73]	Proximity sensor (2)	Eating only Chewing	Ground truth: mark start/end of each eating occasion to the nearest minute Precision: 68% and 83% for general and personalized models Recall: 90% and 93% for general and personalized models	Personalized model had good performance. Socially acceptable design.	3D printed glasses rather than sensor that can be attached to any pair of glasses; those with vision impairment must wear contacts. May falsely classify gum as chewing is a proxy. Soft foods, e.g. yogurt, may be missed. Eating while walking may impact performance. Recharge ∼every 4 h	Free-living 1 day 15 subjects	50 Hz Low-pass filter > gradient > rectification > Teager-Kaiser Energy Operator > Moving average > Thresholding and gap filling
[74]	Proximity sensor (1)	8 foods Chewing	Ground truth: mark occurrence of each chew F1-score: 96.4%	Detect all foods with high accuracy despite different textures. Banana (accuracy 95.14%) scored the lowest	Specialized glasses rather than sensor that can be attached to any pair of glasses; those with vision impairment must wear contacts. Light-based detection so may be impacted outdoors. May be impacted by dentition	In-lab 8 foods 20 subjects	50 Hz 40 features Medium gaussian SVM
[75]	Piezoelectric sensor (1) Accelerometer (1)	Eating only Chewing	Ground truth: mark start/end of each eating occasion to the nearest minute F1-score: 77.7% and 84.9% for general and personalized models	Sensors designed to be attached to any pair of eyeglasses. Small snacks likely to be detected due to 15 s window and utilizing chewing as a proxy	Other chewing habits may be detected, e.g. chewing on straw. Wearing headphones or any device that contacts glasses impacts performance. Less comfortable for users who do not habitually wear glasses	Free-living 1 wk 6 subjects	256 Hz piezoelectric 400 Hz accelerometer 50 features 15 s window DNN
[76]	Accelerometer (1) Camera (1)	Eating Drinking Chewing Head movement Food and beverage type	Ground truth: wearable camera images every 15 s	Combined accelerometer-camera method reduces false positives, e.g. chewing gum from accelerometer or other people’s food from images, and reduces false negatives from accelerometer, e.g. undetected beverage. Sensors designed to be attached to any pair of eyeglasses	Likely energy intensive as camera always on. Image classifier not able to detect foods not previously seen before. Like most methods, does not pick up very small/quick snacks	Free-living 1 d 30 subjects	128 Hz Image-based detection: faster R-CNN Sensor-based detection: 15-layer CNN Random forest to integrate both detections
[77]	OCO sensor (2)—measures skin movement on 2Dplane via optomyography	Eating only Chewing	Ground truth: mark start/end of each eating occasion Precision: 95% Recall: 82% F1-score: 88%	Fast recognition and recognition of small snacks due to use of 4 s window	Specialized glasses rather than sensor that can be attached to any pair of glasses; those with vision impairment must wear contacts. Eating segments contain noneating gestures, e.g. conversation, leading to false negatives	Free-living 2 × ≥8 h per subject 8 subjects	4 s window ConvLSTM
Other devices (*n* = 6)							
[78]	Camera (1) Cap	Eating only HTM motions	Ground truth: video recording	Sensors designed to be attached to any cap. Battery life calculated to be ∼16 h	Must wear cap while eating. False positive when talking, drinking, blowing nose, putting on face mask, mouth rinsing, wiping mouth with napkin, mouth or tongue movement, touching face or mouth	Free-living ≥5 h (2 meals) per subject 10 subjects	30 FPS Each frame classified Offline trained SlowFast model (fusion of two 3D CNN models)
[79]	Accelerometer (1) Gyroscope (1) Magnetometer (1) Optical sensor (1) Gas sensor (1) Air pressure sensor (1) Thermal IR array (1) Time-of-flight ranging sensor (1) Chest-worn badge	Drinking only HTM motions	From confusion matrix, F1-score: 76%, 88%, 81%, 69%, 97% for drinking tea, coffee, milk, water, carbonated water	Optical spectra signal able to distinguish between beverages when consumed in a transparent glass. Eating detection not assessed, but likely can also be detected due to multisensor design	Working current was still >100 mAh, impacting battery life. Activities similar to drinking, e.g. brushing teeth, not assessed.	In-lab 14 activities 10 subjects	All sensors synchronized to 6 Hz MC-CNN
[80]	Accelerometer (1) Gyroscope (1) Back-worn	Eating only Body movement	Ground truth not specified F1-score: 92.68%	Highest performance was for eating classification (F1-score 97.46%). Algorithm installed on wearable, enabling real-time processing. Comfortable to wear. Used LSM9DS1 Inertial Module	Classification between 4 activities only, not reflective of real life behavior. Activities similar to eating, e.g. brushing teeth, not assessed	In-lab 4 activities 5 subjects	30 features 1 s windows CNN
[52]	Piezoelectric sensor (1) Jaw-worn	2 foods (yogurt with apple pieces, croissant) Chewing	Ground truth: video recording F1-score: 94%	LDT0-028K from TE Connectivity Sensors used	Device secured via tape. May falsely classify gum as chewing is a proxy. Soft foods, e.g. yogurt, may be missed	In-lab 5 activities, 17 min per subject 6 subjects	204 Hz 3 s window 29 features SVM
	Respiratory inductance plethysmography (1) 2 belts worn on chest and abdomen	2 foods (yogurt with apple pieces, croissant) Swallowing	Ground truth: video recording F1-score: 58%	RIP sensor from Ben Bulsink Innoveren met Elektronica. Sensor can be positioned beneath clothing	Beverages not assessed and performance may have been impacted by saliva swallows	In-lab 5 activities, 17 min per subject 6 subjects	6.2 Hz 1 breathing cycle = 1 window 28 features SVM
[81]	Accelerometer (1) Gyroscope (1) Forearm	Eating Drinking HTM motions	Ground truth not specified Accuracy: 98.6%	Commercial Myo Armband used.	Armband design not discreet. Foods consumed not specified	In-lab 17 activities 5 subjects	50 Hz 200 ms window with 80% overlap 8 time-domain features for each axis of accelerometer and gyroscope LDA
Multiposition devices (*n* = 5)							
[82]	Accelerometer (5) Gyroscope (5) Magnetometer (5) Both wrists, waist, both ankles	Eating Drinking	Ground truth not specified F1-score: 96.06% From confusion matrix, F1-score: 99.9% and 98.3% for eating with a knife and fork and drinking	5 unobtrusive Xsens DOT IMU devices	Only eating with knife and fork classified, therefore, in addition to exclusion of other utensils, snacks were also excluded	In-lab 13 activities 8 subjects	30 Hz LSTM
[83]	Accelerometer (3) Gyroscope (3) Right wrist, waist, right ankle	Drinking only HTM	Ground truth not specified Accuracy: 99.84% From confusion matrix, 100% for drinking	Latency for recognizing first motion is 2.6 s, and subsequent ones are 1.325 s	More details about the device and experimental setup could be described.	In-lab 13 activities 6 subjects	125 Hz 2.5 s sliding window with 50% overlap CNN
[84]	Accelerometer (3) Gyroscope (3) Both wrists, forehead	Eating Drinking HTM motions Head-to-food motions	Ground truth: video recording F1-score: 61%	Able to capture head-to-food movements (e.g. eating burger) not usually captured in HTM proxies	Sensors not commonly worn on forehead. Study did not utilize commercial device.	In-lab 5 food types, 4 utensil types Water 30 subjects	100 Hz 6 features per IMU MS-TCN
[85]	Accelerometer (2) Gyroscope (2) Dominant wrist, glasses	Meals Drinking HTM motions Head-to-food motions	Ground truth: video recording F1-score: 94.4%	Able to capture head-to-food movements, a common eating style for Asian food, e.g. soup noodles. Commercial Shimmer3 used	One of the Shimmer3 devices were mounted on glasses, which may be less comfortable for users who do not habitually wear glasses. Snacks were not evaluated	In-lab Main meal 24 subjects	128 Hz downsampled to 16 Hz MS-TCN
[86]	Accelerometer (2) Gyroscope (2) Right front trouser pocket, right wrist	Eating Drinking HTM motions	Ground truth not specified F1-score: 99.28% Accuracy: 99.28%	Utilizes commercially available devices	Wearing smartphone on wrist may impact movement. Combination of 5 advanced deep learning models may be computationally expensive	UTwente dataset Each participant contribute ∼30 min data for each activity, including eating and drinking coffee 10 subjects	50 Hz Sliding window with 50% overlap Ens-DeepNet — combo of CNN, LSTM, BiLSTM, GRU, BiGRU

Abbreviations: AFE, analog-front end; ANN, artificial neural network; BiGRU, bidirectional gated recurrent unit; BiLSTM, bidirectional long short-term memory; CNN, convolutional neural network; COCO, Common Objects in Context; ConvLSTM, convolutional long short-term memory; DBSCAN, Density-Based Spatial Clustering of Applications with Noise; DCN-Net, deep convolutional neural network; DNN, deep neural networks; FD, full day; FIC, Food Intake Cycle; FP, false positive; FPS, frames per second; GRU, gated recurrent unit; GWT, Gabor wavelet transform; HMM, hidden Markov model; HTM, hand-to-mouth; IMU, inertial measurement unit; IR, infrared; LDA, linear discriminant analysis; LSTM, long short-term memory; MAE, mean absolute error; MHA, multi-head attention module; ML, machine learning; MS, multistage; O-MCC, optimized multicenter classifier; OREBA, Objectively Recognizing Eating Behaviour and Associated Intake; PPG, photoplethysmography; RBF, radial basis function; RGB, red, green, and blue; RIP, respiratory inductance plethysmography; RMSE, root mean squared error; SE, squeeze-and-excitation; SVC, support vector classifier; SVM, support vector machine; TCN, temporal convolutional network; TP, true positive; TPR, true positive rate; WISDM, WIreless Sensor Data Mining; YOLO, You Only Look Once.

1 The number and type(s) of sensor(s) used. If devices placed on >1 location of the body were used simultaneously in a study, it was summarized under “multiposition devices.” If devices placed on separate locations of the body were compared in a study, they were tabulated separately in their respective locations.

2 The type of intake and eating proxy measured. This includes main meals, snacks, beverages, eating episodes not further defined by authors, and individual foods and/or beverages if a limited number was used and specified in the study.

3 Ground truth method and evaluation metric(s) of sensors. The ground truth is information that is considered real or true. It was used as a comparison against algorithm outputs to evaluate the performance of the device. Any evaluation metric reported either in the text, a table, or a figure that described the performance of the sensor(s) on detecting eating and/or drinking was reported. The best performing method or algorithm result as indicated by the authors was reported for the most realistic setting, e.g. free-living.

4 Advantages and disadvantages of the study, including the study design as well as the device’s feasibility for assisting dietitians in conducting dietary assessments in real-world practice settings. Feasibility was based on the device’s accuracy, social acceptability, real-world and real-time applicability, battery life, and capacity to capture both eating and drinking behaviors.

5 Experiment details including setting, duration, and number of participants. If a paper evaluated the performance of a device in both laboratory and free-living settings, only the results of the free-living setting were presented unless the device was evaluated for different foods in different settings. For example, both settings will be included if the laboratory experiment evaluated beverage intake and the free-living experiment evaluated snack intake. Furthermore, if a study used separate sets to train and validate a device, only the validation set was presented.

6 The data-processing pipeline including algorithm used, sampling rate, and the number of features if available.

#### Wrist-worn devices

Around half of the wrist-worn devices were assessed for their performance in detecting both eating and drinking (*n* = 10/19) [[37], [38], [39], [40],[43], [44], [45], [46],^50^,^55^]. Most were socially acceptable, and some were able to distinguish between utensil [41,50] or food type [55]. Algorithms of some wrist-worn devices have shown the ability to detect meals before their start times by 1 s [51] or even ≤1.5 min [42]. Other algorithms have shown a relatively fast detection speed for eating, such as a 15.97 ms inference time for every 2.2 s of data [47] or a latency of 5.5 s [48].

Disadvantages of devices in this category included overly optimistic results, particularly in studies focused on general daily human activity recognition [37,39,40,[49], [50], [51], [52]]. When measuring intake via hand-to-mouth motions, that is, using the hand to transfer food from the plate to the mouth, foods requiring more head-to-food motions, that is, bringing the head down to the food on the plate, such as sandwiches, burgers, or soup noodles, may be missed. Some devices were only assessed for their performance in detecting either eating [41,42,47,48,[52], [53], [54]] or drinking [49,51] and not both.

#### Neck-worn devices

Neck-worn devices identified in this review utilized sensors such as microphones (*n* = 3), cameras (*n* = 4) and novel sensors such as antennas (*n* = 1), which are likely less sensitive to noneating movement or device placement compared with piezoelectric and proximity sensors. Camera-based devices were able to capture head-to-food movements [58] and food and beverage utensils [60] as they were oriented toward the wearer’s face. Attempts to preserve privacy in such activity-oriented cameras were also made, such as blurring the background and recording only when hand-to-mouth gestures are detected via lower-computation sensors like thermal sensors [62]. Notably, one camera-based device was able to classify eating within the first 1.5 min [60]. Data collected from some microphone-based devices could be distinguished as liquid or saliva swallows [56,59].

According to 1 study, the performance of microphone-based devices could be affected by head movement [57]. In the study using the antenna-based device [61], researchers had to limit data collection to 10-s intervals to prevent fatigue and discomfort from wearing the device. Half of the neck-worn devices were assessed for their performance in detecting both eating and drinking [56,57,60,61], whereas the other half were only assessed for their performance in detecting either eating [58,62] or drinking [59,63]. All but 3 studies in this category used a limited range of foods, utensils, and/or behaviors to train and test their models [56,57,59,61,63].

#### Ear-worn devices

Most ear-worn devices identified in this review were small, discreet, and commercially available. One device featured an algorithm with a 24-s resolution for detecting chewing events [65], whereas 2 others counted individual chews.

User discomfort and the reliance on chewing as a proxy for intake were the main limitations, the latter because of their potential for misclassification of soft foods, omission of beverages, and detection of noneating behaviors such as straw biting and gum chewing. Consequently, none of the identified devices were evaluated for detecting drinking behaviors. Additionally, walking, or other simultaneous activities during eating, may impact chewing identification. Ear-worn devices may also be unsuitable for social eating, especially the use of headphones instead of earbuds [67,70]. Similar to the neck-worn category, all but one study [66] trained and tested their models with a limited range of foods, utensils, and behaviors.

#### Glasses-type devices

All identified glasses-type devices had socially acceptable designs, and most were tested in free-living settings [71,73,[75], [76], [77]]. Half of the glasses-mounted sensors were detachable, allowing them to be affixed to users’ own glasses [71,75,76], and one was a commercially available device [72], reflecting the growing availability of smart glasses [93]. One optomyography-based device, which detected movement of the skin at the cheek and temple, had a fast recognition speed and could detect small snacks using short, 4-s windows [77]. One study had already implemented event-contingent EMA, prompting users to log their intake whenever eating was detected [71].

As for the disadvantages of glasses-type devices, some studies required users to wear a dedicated glasses prototype [31,32,33,36], which could be inconvenient for individuals who regularly wear prescription glasses. Although accuracy was high for most models, it relied on the correct placement and fit of the glasses [30], a finding consistent with the prior scoping review. Glasses-type devices also share similar drawbacks with ear-worn devices related to the use of chewing as a proxy for eating when they use muscle movement of the jaw to activate the camera on the glasses. The algorithm of only 1 device was evaluated for its performance in detecting both eating and drinking [76]; all other glasses-type devices were evaluated for eating only [[71], [72], [73], [74], [75],^77^].

#### Other devices

Six devices were categorized as “other”—a cap-mounted camera [78], a multisensor, chest-worn badge [79], an accelerometer and gyroscope worn on the lower back [80], a jaw-mounted piezoelectric sensor [52], respiratory inductance plethysmography belts worn around the chest and abdomen [52], and a forearm-worn accelerometer and gyroscope [81]. Only the cap-mounted camera was assessed in free-living settings for ≥5 h per subject. However, this device may not be appropriate for use in formal dining settings. Devices in this category featured rudimentary or cumbersome designs, such as the jaw-mounted sensor being secured by tape or the bulky design of the chest-worn badge and forearm-worn sensor. The algorithm of only 1 device was evaluated for its performance in detecting both eating and drinking [81]; all other devices were evaluated for either eating [52,78,80] or drinking only [[71], [72], [73], [74], [75],^77^,^79^].

#### Multiposition devices

Five multiposition devices were identified, all of which utilized accelerometers and gyroscopes. One study positioned them on both wrists, both ankles, and the waist [82]; another on the right wrist, right ankle, and the waist [83]; a third on both wrists and the forehead (secured via a headband) [84]; the fourth on the dominant wrist and glasses [85], and the last on the right wrist and right front trouser pocket [86]. Multiposition devices incorporating sensors worn on or near the head have the advantage of capturing head-to-food movements [84,85], which is particularly beneficial for identifying the consumption of Asian dishes such as soup noodles [85]. One study [83] also reported a short latency period for recognizing eating gestures, with 2.6 s for the first motion and 1.325 s for subsequent motions.

Along with the user discomfort noted in the prior scoping review, other disadvantages of devices in this category included their placement on unconventional body locations, such as the forehead. The algorithms of 4 devices were evaluated for their performance in identifying both eating and drinking behaviors [82,[84], [85], [86]], whereas the algorithm of one device was only evaluated for its performance in identifying drinking behaviors [83]. All devices were assessed in stringent, in-lab settings with a limited range of foods, utensils, and behaviors.

**FIGURE 2:**
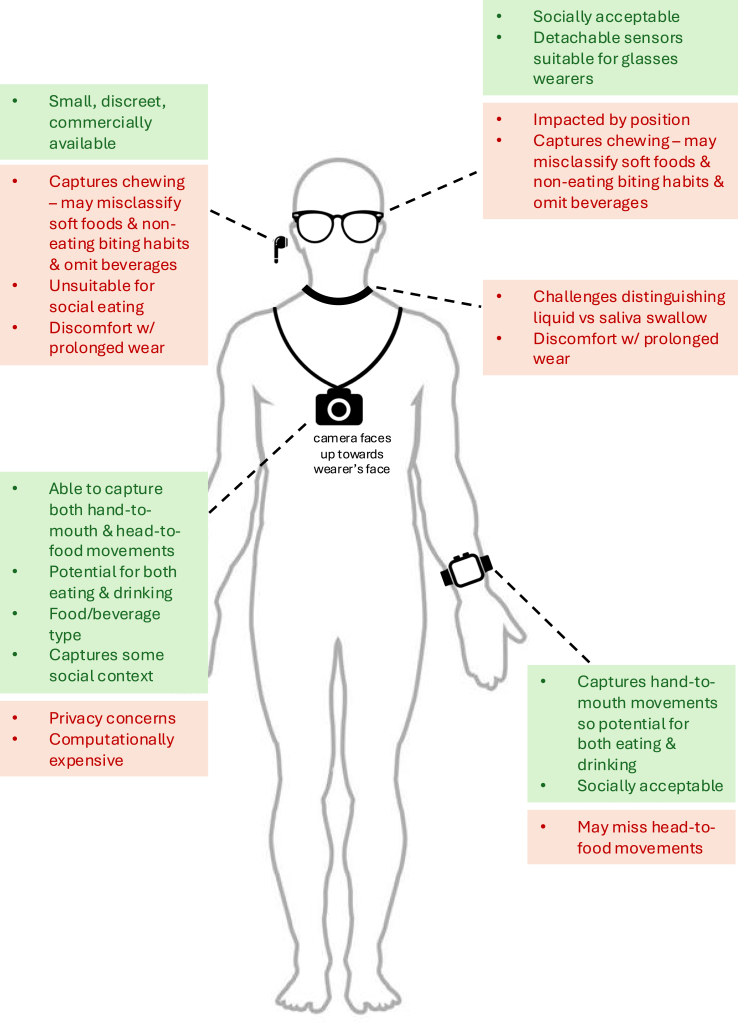
An overview of single-sensor categories, showing their body placement and highlighting key strengths and weaknesses within each category.

Figure 2 provides a visual representation of the single-sensor categories, showing their body placement and highlighting some key strengths and weaknesses within each category.

## Discussion

This scoping review on sensor-based eating detection provides an update to a previous review published in 2022 [10], capturing an additional 52 devices designed to passively detect eating and/or drinking in real time. Identified devices were assessed against 6 feasibility criteria—accuracy ≥80%; evaluation in settings where subjects were free to choose their own foods and activities; social acceptability and comfort; long battery life; real-time detection of eating episodes; and the ability to detect both eating and drinking—to evaluate their suitability for real-world dietary assessment. As in the prior review, none of the identified devices fulfilled all feasibility criteria. However, the prior review only applied 5 criteria, as the ability to detect both eating and drinking was not required [10]. Compared with the prior review, this update saw a greater consideration of socially acceptable device designs, as well as the computational burden and battery consumption of model processing. As a result, had the sixth criterion (both eating and drinking detection) not been added in the current review, 2 devices—1 glasses-type device [75] and 1 neck-worn camera [62] would have met all 5 original criteria.

The glasses-type device that met the original 5 feasibility criteria incorporated a piezoelectric sensor and an accelerometer to collect chewing signals from temporalis muscle movement and mechanical vibrations [54]. Its accurate performance was attributed to model training using participants’ own data—an approach that, although time intensive, has become more prevalent [48,54,64,73,75]. Additionally, its short 15-s detection window and reliance on a chewing proxy rather than hand-to-mouth movements suggest improved detection of small, quick snacks. Its inability to detect drinking events could potentially be addressed by integrating a microphone to capture beverage swallowing sounds and an on-device camera to automate the capture of dietary intake, such as that of Automatic Ingestion Monitor Version 2 device [13]. This setup could further benefit dietary assessment by enabling the passive capture of dietary intake, removing the need for participant input of data or image taking.

The neck-worn camera device incorporated an accelerometer to verify whether the device was being worn, and a thermal sensor to identify hand-to-mouth gestures, which subsequently activated the onboard, egocentric camera for recording [62]. This noncontinuous capture approach not only conserved battery life but also enhanced user privacy, thereby improving social acceptability. Additional privacy-preserving features included background blurring and the use of activity-oriented or egocentric cameras, which have seen notable advancements in recent years [58,60,63]. Although the device demonstrated high accuracy during offline testing, real-time performance was comparatively lower due to reliance on the thermal sensor, which was employed to reduce power consumption. This reduction in accuracy is likely attributable to the thermal sensor’s susceptibility to ambient temperature fluctuations [62,94]. Although the device does not yet meet all 6 feasibility criteria, it shows promise, as hand-to-mouth gestures, common to both eating and drinking, serve as a viable proxy for intake detection.

Compared with the prior review [10], the updated search also revealed broader research on daily human activity recognition [[37], [38], [39],^79^,^80^,^82^,^83^,^86^] and a greater use of large, publicly available datasets of intake gestures with synchronized sensor data and annotations [40,42,45,47, 48,53,85]. Additionally, there was a greater emphasis on the ongoing development and enhancement of existing prototypes rather than the proposal of new ones [9], as evidenced by the use of the same 6 categories of devices from the prior review, with no new major device types emerging. Although wrist-worn devices remained the most popular hardware category in this updated review, our findings suggest that smart glasses are emerging as the next major advancement in wearable technology [95]. Glasses-type devices have undergone substantial development in recent years, with many of the studies in this updated review building upon those in the prior scoping review [[71], [72], [73], [74],^76^]. Products such as the Ray-Ban Meta eyewear are already commercially available [96], and the integration of smart glasses in healthcare settings continues to expand [97,98]. This growing trend underscores the potential of smart glasses to become a key tool in revolutionizing both dietetic practice and research, provided that clients are willing to adopt the technology.

However, as the use of wearable cameras becomes more mainstream with the proliferation of consumer-grade, camera-embedded smart glasses, their social acceptability must be examined, not just in terms of physical design [99], but also in relation to privacy and ethical considerations. The newer, less obtrusive generation of camera-embedded smart glasses with discreet interaction features can capture photos and videos of bystanders without their knowledge [100]. Wearers of commercial smart glasses consider the currently available privacy indicators ineffective [100], as gestures such as raising a hand to press the image capture button, touchless interaction such as voice commands, and small LED lights to indicate recording [96] are much less obvious than image capture or recording with a smartphone [101]. One study showed that individuals who did not own smart glasses expressed stronger concerns about the technology’s appropriateness and safety in public settings, particularly regarding privacy, antisocial behavior, and potential harm, than those who owned the device [102]. These individuals also more strongly believed that wearing such devices could be considered disrespectful [103,104], inappropriate, or offensive [102], ultimately reducing their social acceptability [103,104]. In addition to the potential negative implications for bystanders, previous research has documented how such tensions can manifest in negative reactions from bystanders [105]. Wearers of earlier, more conspicuous smart glasses were sometimes verbally and physically attacked due to the assumption that they would record videos without consent [100]. Furthermore, connected smart glasses also pose potential security threats to both users and bystanders, including risks of video hijacking, surveillance, and identity theft [106]. In contrast, neck-worn wearable cameras, particularly those designed for egocentric or activity-oriented monitoring, may help mitigate some of these limitations. These devices are typically more visible and face the user, reducing the likelihood of inadvertently capturing bystanders. Recent iterations also incorporate privacy-preserving features such as automatic background blurring and event-triggered recording based on detected eating gestures [62], which limit continuous video capture. Similarly, smart glasses that passively trigger cameras via sensors, such as those detecting eating gestures to identify eating events, may also help mitigate some of these issues by reducing continuous recording of passersby [13,75]. However, when recording is active, clearer and more visible indicators should be incorporated into the device design to ensure transparency. Progress in this space should move beyond a predominantly consumer-centric approach focused on overcoming adoption barriers, such as concealing cameras, and instead address broader social needs, including clearer ways of informing people when they are being captured or recorded [107].

This review also captured several devices that had the potential to trigger the delivery of JIT interventions or event-contingent EMA that can prompt individuals to log or capture an image of their intake [11,12]. Most existing JIT interventions are either time-based, such as delivering breakfast-related interventions in the morning [108], or rely on participant-reported data, such as responses to signal-triggered EMA indicating high dietary lapse risk [109] or discrepancies between self-reported intake and dietary goals [22]. Meanwhile, most event-contingent EMA rely on the memory of the participant to initiate the reporting of the eating event themselves [110]. In contrast, the devices reviewed here can enable true real-time interventions or device-triggered, event-contingent EMA as intake is continuously, rapidly, and automatically detected. For example, the algorithms of 2 wrist-worn devices were able to detect meals before their start times by 1 s [51] or even ≤1.5 min [42]. Other wrist-based algorithms have shown a relatively fast detection speed for eating, such as a 15.97 ms inference time for every 2.2 s of data [47] or a latency of 5.5 s [48]. One wrist-worn device also showed the ability to monitor eating speed [53], which alone could inform a JIT intervention to encourage slower eating. These attributes are also reflected by the ear-worn devices; 1 device featured an algorithm with a 24-s resolution for detecting chewing events [65], whereas 2 others counted individual chews, offering potential for monitoring eating speed as well [66,68]. An eyeglass-based device [75] demonstrated the ability to capture small snacks through a 15-s detection window, which could also be particularly beneficial for populations with irregular eating patterns, such as young adults [111]. One study has already demonstrated the effective implementation of sensor-based, event-contingent EMA [71]; when eating was detected 5 consecutive times, notifications were sent to the user’s smartphone application reminding them to log their intake. If the user was not eating, they selected “no” on the application. Whether a device is more suitable for JIT intervention or event-contingent EMA depends partly on the algorithm’s performance—a higher sensitivity (i.e., fewer false negatives) may be preferable for delivering event-contingent EMA, prompting users to confirm and report their eating occasions. In contrast, a higher precision (i.e. fewer false positives) may be more suitable for JIT interventions to minimize unnecessary disturbances [43].

N-of-1 study designs can also benefit from incorporating sensor-based detection of eating behaviors. Sensor-based devices that continuously and rapidly detect eating behaviors offer improvements over self-reported methods by increasing data granularity, capturing more variations in eating patterns, and reducing both recall bias and participant burden, common challenges in repeated measurement protocols [24]. The lower burden also likely allows these methods to be sustained over longer periods, enabling even greater data granularity. For example, a chewing-detection device could be used in an observational N-of-1 trial to evaluate the effects of eating speed [53] and meal timing on satiety, energy levels, or eating contexts. In such a setup, the real-time detection of chewing could prompt event-contingent EMA to assess mood or hunger [112], or trigger wearable cameras to capture contextual images reflecting social and environmental influences [29]. When deployed continuously throughout the day, a more accurate and nuanced representation of an individual’s behaviors can be captured. These data can not only support N-of-1 study designs but also serve as a success indicator for individuals engaging in self-monitoring and self-experimentation. This may include testing personalized strategies such as reducing snacking or improving mindfulness through longer meal durations or slower eating speeds. Combined with other health markers such as body weight or blood glucose levels, this approach can improve disease risk estimation [113] and establishing more precise diet–disease relationships.

The large amounts of individual-level data gathered in N-of-1 trials from wearable sensors (for example, eating speed and timing) and other data sources along with the capacity for these devices to trigger JIT interventions can serve as a foundation for, or enhance the effectiveness of, precision nutrition [24]. Precision nutrition relies on integrating diverse and variable data types on a single individual to design tailored dietary interventions. This may include the analysis of complex gene–environment interactions and deep phenotyping, as well as the screening and incorporation of behavioral and sociocultural factors, such as dietary habits and environmental influences, and health characteristics [114]. Wearable sensors can help trigger these tailored interventions moments when individuals are most receptive, such as during eating or meal preparation, improving both the relevance (tailored interventions) and the effectiveness (timely interventions) of dietary strategies [115]. Further research is warranted to explore the application of these technologies in this area.

A limitation of this scoping review was the reporting of the “best” evaluation metric of each study, albeit only in the most realistic settings. For example, if a study included both laboratory and free-living performance metrics, we reported only the free-living results, even if laboratory performance was higher. In addition, our social acceptability feasibility criterion was primarily based on the appearance and description of the device prototype, as judged by the researchers. This approach may not fully capture privacy and ethical concerns, particularly for sensor-based devices with wearable cameras. Furthermore, social acceptability is highly context-dependent and can vary across cultures and settings, and this assessment may be limited by researcher bias. A major strength of this review was that it was written with limited jargon by Accredited Practising Dietitians with experience in the Nutrition Care Process and dietary assessment. The feasibility criteria used to assess devices’ suitability for dietetic practice settings ensure this review is highly relevant and practical for a dietetic audience. The systematic process of the PRISMA-ScR framework guided this review, ensuring replicable, transparent, and comprehensive results. The wide scope of the search (Supplementary Material) and inclusion of both published and unpublished literature further maximized the capture of relevant literature, providing a comprehensive overview of this rapidly developing field.

This scoping review update highlighted substantial advancements in the development of sensor-based devices for detecting eating and drinking over the past 4 y, particularly in the realm of glasses-type devices. These devices are becoming increasingly applicable for use in real-world dietetic practice settings, based on the feasibility criteria outlined in this review. This suggests that dietetic practice methods are on the verge of being revolutionized, with the potential for more passive and automated dietary intake collection in the near future.

## Author contributions

The authors’ responsibilities were as follows – AR, MA-F, EH: designed research; LW: conducted research; LW, AR, MA-F: wrote paper; AR: had primary responsibility for final content; and all authors: read and approved the final manuscript.

## Data availability

Data described in the manuscript, code book, and analytic code will not be made available because no primary data were generated.

## Declaration of generative AI and AI-assisted technologies in the writing process

During the preparation of this work, the author(s) used ChatGPT (OpenAI) to assist with wording, grammar and structure. After using this tool/service, the author(s) reviewed and edited the content as needed and take(s) full responsibility for the content of the publication.

## Funding

This work was supported by an Ignition Grant (grant number: A-5575940775) from a University of Sydney and University of California San Diego partnership.

## Conflict of interest

The authors report no conflicts of interest.
