# Supplementary material for: A Scoping Review of Sensor-Based Capture of Eating and Drinking Occasions That Could Be Used for Enhancing Personalized Nutrition Interventions in Real Time
Source: Adv Nutr. 2025 Dec 20;17(2):100575. doi: 10.1016/j.advnut.2025.100575 (PMC12887789; doi:10.1016/j.advnut.2025.100575)
Supplement: multimedia component 1 [file mmc1.docx]

**Supplementary Material**

Technology-based dietary assessment tools – MEDLINE Search Strategy (Literature Search performed: 5 September, 2025)

1. Nutrition Assessment/
2. Nutrition Surveys/
3. 1 or 2
4. “Diet, Food, and Nutrition”/
5. Eating/
6. 4 or 5
7. 3 and 6
8. Eating.tw.
9. Dietary behavio?r.tw.
10. Feeding.tw.
11. “Dietary intake”.tw.
12. “Food intake”.tw.
13. 7 or 8 or 9 or 10 or 11 or 12
14. Monitoring, Ambulatory/
15. Wearable Electronic Devices/
16. Mobile Applications/
17. Smart Glasses/
18. Internet-Based Intervention/
19. “Ambulatory monitoring”.tw.
20. “Electronic health”.tw.
21. “Mobile health”.tw.
22. smart?watch.tw.
23. wearable sensor*.tw.
24. wearable device*.tw.
25. “Wearable camera”.tw.
26. “Ecological momentary assessment”.tw.
27. 14 or 15 or 16 or 17 or 18 or 19 or 20 or 21 or 22 or 23 or 24 or 25 or 26
28. Time Factors/
29. (“early” or “earlier” or late* or hour*).tw.
30. (time or timing or schedul* or pattern* or variability or variation or detect* or identif* or recogni* or chang*).tw.
31. 28 or 29 or 30
32. 13 and 27 and 31
